# Abnormal Apoptosis of Trophoblastic Cells Is Related to the Up-Regulation of CYP11A Gene in Placenta of Preeclampsia Patients

**DOI:** 10.1371/journal.pone.0059609

**Published:** 2013-03-29

**Authors:** Guolin He, Wenming Xu, Yan Chen, Xinghui Liu, Mingrong Xi

**Affiliations:** 1 Department of Obstetrics and Gynecology, West China Second University Hospital, Sichuan University, Chengdu, China; 2 Joint Laboratory for Reproductive Medicine, Sichuan University-The Chinese University of Hong Kong (SCU-CUHK), West China Second University Hospital, Sichuan University, Chengdu, China; 3 Key Laboratory of Obstetric & Gynecologic and Pediatric Diseases and Birth Defects, Ministry of Education, West China Second University Hospital, Sichuan University, Chengdu, China; Yale School of Public Health, United States of America

## Abstract

Abnormal placenta trophoblast proliferation and apoptosis is related to the pathogenesis of preeclampsia. Emerging evidence has also indicated that key pregnancy-associated hormones, such as hCG, progesterone, are found in high concentration at the maternal-fetal interface. The purpose of this study was to investigate the expression of CYP11A, a key enzyme in steroid hormone synthesis and metabolism, in normal pregnancy and severe preeclampsia placenta and to explore the underlying mechanism of the relationship between the altered CYP11A expression and onset of preeclampsia. Immunohistochemistry method was used to study the localization of CYP11A-encoded protein P450scc in the placenta; reverse transcription polymerase chain reaction (RT-PCR) and Western blotting were used to examine CYP11A expression at mRNA and protein levels in patients with severe preeclampsia and normal placental tissue. CYP11A overexpression in trophoblastic cells was used to evaluate the effect on viability. TUNEL staining was used to determine whether overexpression of CYP11A could affect trophoblastic cell apoptosis. The results showed that CYP11A was selectively expressed in the cytoplasm of the placental trophoblastic cells. CYP11A expression were significantly increased in severe preeclampsia compared with normal pregnancy in both mRNA and protein levels. Multiple regression analysis indicated that CYP11A gene expression was positively correlated to ALT level and Plt, while negatively correlated to INR. Overexpression of CYP11A reduced trophoblastic cell proliferation and induced HTR8/SVneo cells apoptosis through activation of activated caspase-3 expression. These results suggest that abnormally high expression of CYP11A inhibits trophoblastic proliferation and increases apoptosis and therefore could be involved in the pathogenesis of preeclampsia.

## Introduction

Preeclampsia is a serious pregnancy-related complication with an incidence of approximately 5∼6% in pregnant women [1]. The main clinical manifestations include hypertension, proteinuria, edema and systemic dysfunction of multiple organs. Although the etiology of preeclampsia remains poorly understood, mounting evidences have indicated that genetic factors contribute to the etiology of preeclampsia. Previous studies have proposed hundreds of candidate susceptibility genes from genetic association analysis, yet so far no consensus exists to the identity of the preeclampsia susceptibility gene [2]–[3] and the exact role of these genes in the pathogenesis of preeclampsia is far from clear [4]–[7].

One important characteristic of preeclampsia is that when the placenta leaves the mother, such as in the termination of pregnancy, symptoms of preeclampsia are alleviated. Termination of pregnancy is so far the only effective and thorough treatment [8]. In some cases of postpartum eclampsia, namely postpartum eclampsia with residues in the uterus, the symptoms can be relieved upon complete removal of residual placenta [9], supporting the prevailing view that the placenta factors contribute the major source of the disease risk. However, the exact mechanism of how the placenta factors cause symptoms of multiple organs is still poorly understood. Placenta microarray studies have helped to identify the true preeclampsia susceptibility genes from case-control study. So far 13 microarray studies have been used to explore placenta gene expression from preeclampsia patients, and six of which have examined placenta gene expression at the whole genome level [10]–[20]. Enguobahrie DA et.al used placenta microarray to screen for preeclampsia candidate genes and suggested that although some differentailly expressed genes may potentially be related to the onset of preeclampsia, such as the cholesterol side-chain cleavage enzyme gene CYP 11A).

CYP 11A encodes a cholesterol side chain cleavage enzyme (cytochrome P450 cholesterol side-chain cleavage, P450scc), which catalyzes the cholesterol side chain split, and the transformation of pregnenolone, a precursor for all other steroid hormones. During pregnancy, the placenta synthesizes a large number of steroid hormones, which are closely related to start and maintenance of pregnancy, fetal development and delivery. CYP11A is expressed in placenta and responsible for the placenta-derived hormone synthesis [21]. On the other hand, the process of embryo implantation and placental formation depend on a series of events including cell proliferation, invasion and apoptosis, as well as cell-cell recognition and interaction between the trophoblast and endometrium. The abnormal proliferation and apoptosis of the placental trophoblast cells have been shown to be important characteristics of preeclampsia pathology. Interestingly, the CYP 11A gene is involved in cell growth, proliferation, and differentiation in a variety of tissues, suggesting that this gene may be one of candidate preeclampsia susceptibility genes [22]. Therefore, we proposed that CYP11A might be involved in the pathogenesis of preeclampsia via regulating trophoblastic cell proliferation and apoptosis. This study was designed to explore the relationship between the CYP11A gene and preeclampsia and the role of CYP 11A gene in proliferation and apoptosis of trophoblast cells.

## Materials and Methods

### Sample Choose Criteria

All the subjects in this study attended our hospital between March 2007∼May 2009 for cesarean section at gestational weeks between 35 weeks and 39 weeks. 53 cases of normal pregnancy, 37 cases of severe preeclampsia and 22 cases of mild preeclampsia were recruited for the study. The study was approved by the ethics committee of West China Second University Hospital of Sichuan University and informed consent have been obtained from all the subjects prior to the study. Pregnant women are informed about the potenial risks and benefits to themselves, the foetus and their subsequent offspring, as well as their fertility, in accordance with the 2002 International Ethical Guidelines for Biomedical Research Involving Human Subjects of the Council for International Organizations of Medical Sciences (CIOMS). Preeclampsia was defined as having a systolic blood pressure of >140 mmHg and/or diastolic blood pressure of >90 mmHg on two occasions >6 h apart after 20 weeks of gestation, while before the onset of labor, plus proteinuria of >2+ (dipstick method) or >0.3 g/24 h (American College of Obstetricians and Gynecologists, 1996). Severe preeclampsia was defined as having a higher blood pressure >160 mmHg systolic or >110 mmHg diastolic on two occasions >6 h apart, and a proteinuria level >5 g/24 h or >3+ by dipstick testing on at least two separate occasions (American College of Obstetricians and Gynecologists, 1996). The gestation-matched placentas of normal pregnancy were chosen as normal control. All the subjects were singleton pregnancies without previous heart, liver, kidney, thyroid, or diabetes histories.

### Specimen Collection

The placentas were collected after cesarean section. Tissues around the central villus area were isolated, rinsed with saline. The tissues for immunohistochemical staining were fixed in 4% PFA. For the RNA and protein extraction, tissues were cut into 1*1*1 cm in size and freezed in liquid nitrogen.

### Immunohistochemistry

Immunohistochemical staining was carried out according to conventional operation of immunohistochemistry kit (Histostain™-Plus Kits, Cat.Number: SP-9000; Beijing Zhongshan Biotechnology Co., Ltd) with minor revision. Briefly, the tissues were embedded in paraffin blocks. 5-mm sections were sequentially cut and mounted onto gelatin-coated slides. Then they were dried overnight at 37°C. After that, all slides were deparaffinized in xylene and rehydration through graded ethanol. In order to retrieve the epitope, slides were immersed in citrate buffer at pH 6.0 in a boiling water bath for 20 minutes. The slides were cooledat room temperature for 20 minutes, and then incubated in H_2_O_2_ (0.3%) at room temperature for 20 minutes. After serum blocking, slides were incubated with CYP11A antibody (l:200,Boster, Wuhan, China) at 4°C overnight. PBS instead of primary antibody was used as the blank control. 2^nd^ Antibody and DAB reagents were used from the Histostain™-plus kit (Beijing Zhongshan Biotechnology Co., Ltd,Beijing,China) Immmuohistochemical results were analysed by light microscopy (TI-U,Nikon CLEIPSE,NIKON, Japan) equipped with camera (SPOT FlexTM Camera, Diagnostic Instrument, USA). The software for image analysis was SPOT advance.

### Reverse Transcriptase Polymerase Chain Reaction Method (RT-PCR, Reverse Transcription PCR)

Total RNA was extracted from placenta in strict accordance with the instructions of the manufacturers of the TRIZOl (, Invitrogen, USA). RNA concentration were measured and adjusted to 1 ug/ul and stored at −80°C. The cDNA synthesis reaction was as the follows: total RNA 12 ul were mixed with Oligo (dT) 1.5 ul and DEPC-treated water 4.5 ul and placed at 70°C for 5 min to denature the template. The reaction was quickly placed on ice for l min and the following reagents added: 5 × buffer 6 ul, 10 mM of dNTP 3 ul, 25 U/ul RNase in l.5 ul, 200 U/ul reverse transcriptase enzyme 1.5 ul. The reaction was incubated at 42°C reaction for 60 min, 95°C for 5 min to inactivate reverse transcriptase, and on ice for 5 min to complete degradation of RNA. Primer sequences for CYP 11A were (forward) 5′-TATGTC CAT CGACCC TGAAGA -3′ and (revserse) 5′-GGA ACA GGTCTGGGGGAA G–3′. The length of PCR product of CYP11A was 469 bp. The primer sequences for GAPDH were: (forward)5′-CTTAGCACCCCTGGCCAAG -3′ and (reverse) 5′-GAT GTT CTG GAG AGC CCC G CCC G-3′. The length of PCR product of GAPDH was 450 bp. Amplification conditions: denature at 95°C for 4 min, 25 cycles of denature at 95°C for 1 min, annealing at 50°C for 45 sec, extension at 72°C for 45 sec, followed by final extension at 72°C for 7 min. The PCR products were subjected to electrophoresis and stained with ethidium bromide. The intensity of bands was analyzed with Quantity One software (Bio-Rad, USA). The corresponding internal reference GAPDH was used as loading control.

### Western Blot

Placenta tissue or HTR-8/SVneo cells were lysed in RIPA buffer (150 mM sodium chloride; 1.0% NP-40 and Triton X-100; 0.5% sodium deoxycholate; 0.1% SDS; 50 mM Tris, PH8.0) Whole cell protein was extracted by centrifuge at 12000 g at 4°C for 30 min. BCA method was used to quantify the protein concentration. Proteins were separated by SDS-PAGE and blotted onto nitrocellulose membrane. The blots were incubated with rabbit anti-human CYP11A antibody (1∶200, Boster, Wuhan, China) or cleaved caspase-3 antibody (1∶1000, Cat:1476-1,Epitomics, Burlingame,USA) at 4°C overnight and HRP-conjugated goat anti-rabbit IgG at room temperature for 1 hour (1∶5000, KPL Inc, China). The blots were detected using chemiluminescence kit (Cat. Num:WBKLS0500, Millipore company,USA) and the signal was developed in the gel imaging system. The intensity of bands was analyzed using Quantity One software (Bio-Rad,USA). The corresponding internal reference GAPDH (1∶3000, Kangchen BIO-TECH Corporation, Shanghai, China) or beta-tubulin (Zen BioScience, Chengdu, China) were used as internal controls.

### Cell Culture and Transfection

Human extravilloustrophoblast (EVT) cell line,HTR- 8/SVneo, were cultured in RPMI 1640 medium supplemented with 10% fetal bovine serum at 37°C in a 5% CO_2_ atmosphere. CYP11A cDNA was cloned from human testis cDNA and inserted into the pCDNA3.1 plasmid with BamH1 and ECoR1 restrictive enzyme sites. The correct insert of CYP11A cDNA in pcDNA-3.1-CYP11A was confirmed by sequencing. The CYP11A overexpression plasmids (pcDNA-3.1-CYP11A) were transfected into HTR-8/SVneo cells with lipofectamine™ 2000 reagent following the protocol of Invitrogen. For culture under hypoxic conditions, cells were cultured in a humidified chamber (MCO-5M, SANYO,JAPAN) that was continuously injected with a mixture of 5% CO_2_, 2% O_2_ and 93% N_2_ for 72 hr. Cells were cultured in serum-free medium containing 5 mg/ml BSA in 96-well microtiter plates for hypoxic treatment.

### Cell Viability and Apoptosis Assay

1000 cells were seeded to each well of microtiter plates in an atmosphere containing either 20% or 2% O_2_. For Giemsa staining of cell, 150 µl of giemsa reagent was added for 30 min. After PBS wash, the absorbed stain was then solubilized with 150 µl of methanol and the optical density was measured at 650 nm using Microplate Reader (iMarkTM,Bio-RAD, USA). Apoptosis was detected by the terminal deoxynucleotidyl transferase-mediated dUTP nick end-labeling (TUNEL) method using FragEL™ DNA Fragmentation Detection Kit (Cat.Number: QIA39, Millipore, Germany) following instruction. The percentage of TUNEL labeled cells was determined from 200 cells.

### Statistical Analysis

All the data were presented as mean+/−SD unless specified. SPSS 15.0 statistical package was used for statistical analysis. The F-test of two-sample homogeneity of variance was used to analyze the homogeneity of the samples. Student t-test and variance analysis were used for the comparison between two groups and three groups. A probability (P)<0.05 was considered statistically significant. Correlation analysis was used to determine the relationship betweenCYP11A expression and clinical parameters. The partial correlation analysis was used to determine the independent correlation relationship between the CYP11A expression level and clinical parameters.

## Results

### Comparison of the Clinical Features of the Preeclampsia Groups and the Normal Pregnancy Group

The clinical data of mild, severe preeclampsia and normal pregnancy are shown in Supplemental Table S1 and S2. As shown in Table S1, there were no significant differences relating to age and motherhood among the three groups. However, the gestational age of patients in the severe preeclampsia group was significantly lower compared with those in the mild preeclampsia and normal pregnancy group. As shown in Table S2, there was a significant difference in the following clinical parameters in each group: the incidence of gestational age, weight gain during pregnancy, before delivery, gestational age, blood pressure and its changes, liver, renal function and neonatal weight.

### Expression and Localization of P450scc in Trophoblast

**Figure 1:**
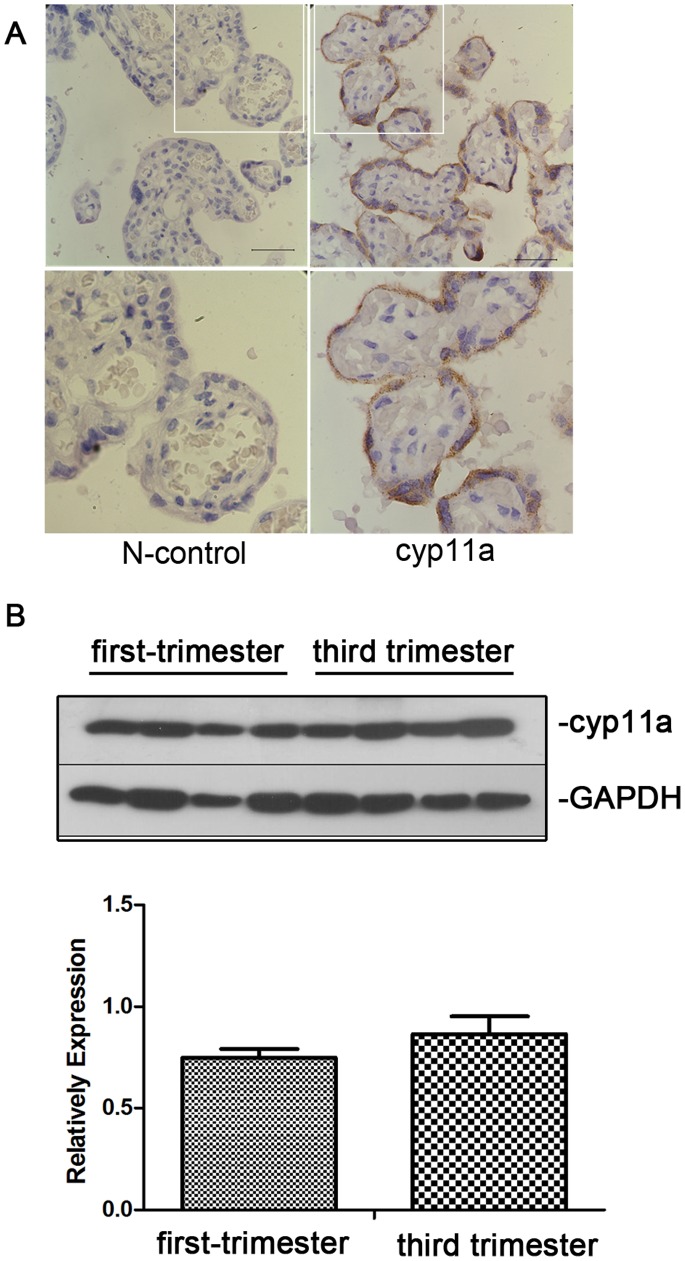
Immunohistochemistry staining of P450scc in placenta tissue. (A) Upper panel: Visible P450scc positive expression located in the cytoplasm of syncytiotrophoblast of placenta (arrow). Left: negative control without primary antibody. Right: P450scc (CYP11A) staining; Manification 600x; scale bar = 40 uM. Down panel: the higher manification pictures of the insert part of the above figures (B) Western blot shows that there is no significant different of CYP11A expression between first and third trimester placentas.

We examined the expression and localization of P450scc protein in placenta by immunohistochemistry. The result showed that P450scc was expressed in the trophoblasts of the placental tissue at third trimester. P450scc were mainly located in cytoplasm of syncytiotrophoblast cells (Fig. 1), which was in agree with the previous report that P450scc islocated in mitochondrial of trophoblast cells. We also examined the developmental changes in CYP11A expression during pregnancy from placenta of first trimester to third trimester pregnancy by Western blot. Our results showed that there was no significantly difference between first trimester placenta and third trimester placenta in P450scc protein expression, suggesting that that there is no significant changes in P450scc protein expression during normal placenta development (Fig. 1B).

### CYP 11A Gene mRNA Expression Levels in Normal Pregnancy and Preeclampsia Placentas

We then examined whether CYP11A expression was altered in preeclampsia placenta. As shown in Table 1, CYP11A mRNA expression was significantly higher in preeclampsia placenta (ROD = 1.1746±0.7252) compared to that of normal pregnancy group (ROD value equals 0.8646±0.2954, P<0.05). CYP11A mRNA expression levels in the severe preeclampsia group was significantly higher than the normal control group (ROD value 1.1964±0.7024 vs. 0.8646±0.2954, P = 0.015). However, there was no significant difference between the severe preeclampsia group and the mild preeclampsia group (ROD value 1.1964±0.7024 vs. 1.0939±0.8398 P = 0.727) or between the mild preeclampsia group and normal pregnancy group (ROD value 1.0939±0.8398 vs. 0.8646±0.2954, P = 0.241). These results indicate that CYP11A is upregulated in preeclampsia.

**Table 1:** of CYP 11A mRNA in normal pregnancy group, severe pre-eclampsia group compared with the relative optical density of expression in mild preeclampsia.

Grouping	The number ofcases	ROD
Normal pregnancy group	59 59	0.8646±0.2954
Severe preeclampsia group	37 37	1.1964±0.7024
Mild preeclampsia	22 22	1.0939±0.8398

Normal pregnancy group with severe preeclampsia group: p = 0.015,95% CI (−0.5388, −0.0595).

Normal pregnancy group compared with mild preeclampsia: p = 0.241,95% CI (−0.6510, 0.1565).

Mild preeclampsia and severe preeclampsia group: p = 0.727,95% CI (− 0.4656, 0.3259).

### P450scc Protein Expression in Placenta of Normal Pregnancy and Preeclampsia Patients

**Figure 2:**
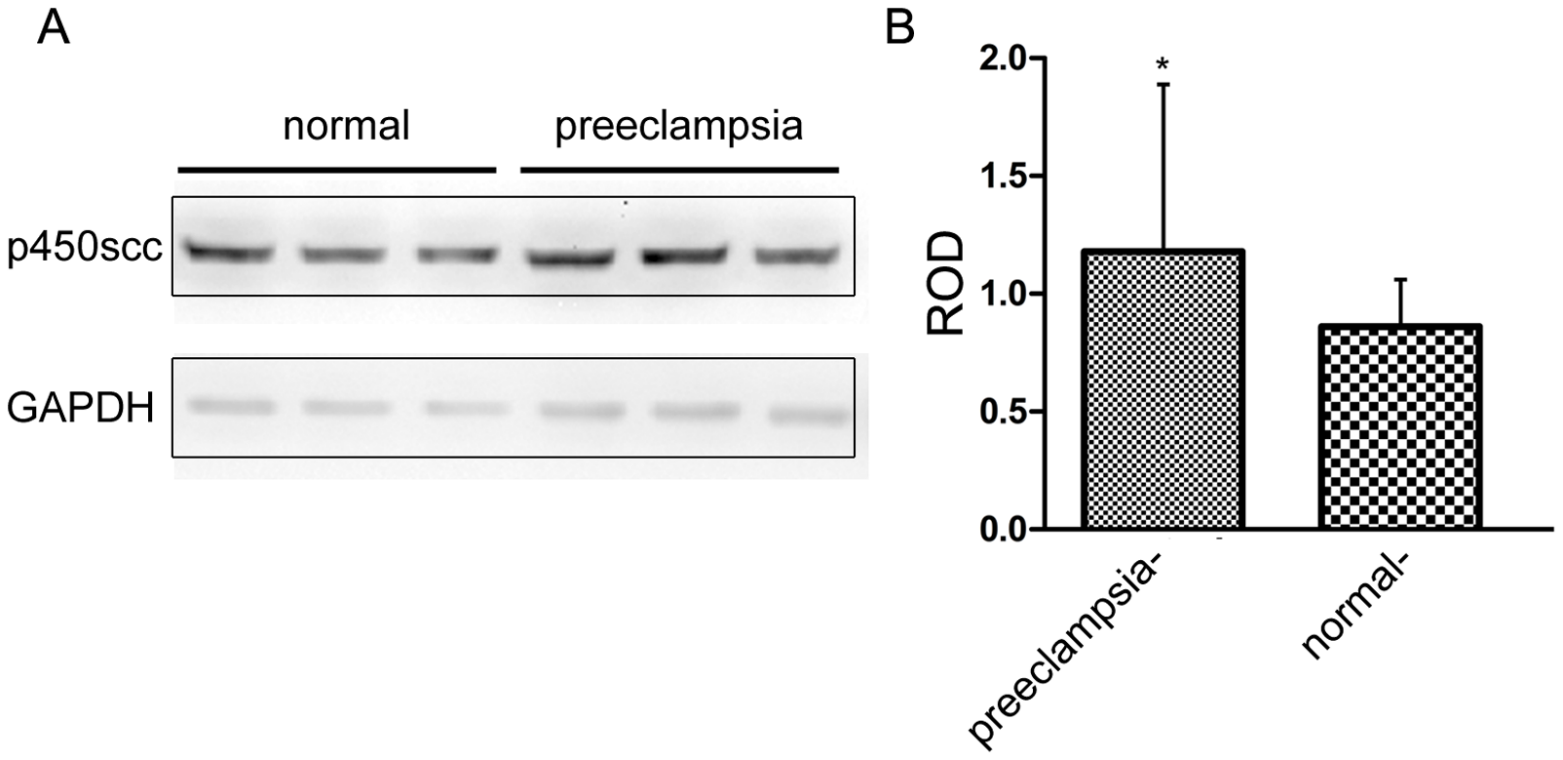
P450scc protein expression in preeclampsia group was significantly higher than normal control. Left panel: representative western blot result of the placenta samples, Right panel: statistic of western blot result from 53 normal samples and 59 preeclampsia samples (p<0.05).

Western blot result shows CYP 11A gene encoding P450scc protein with a relative molecular mass of 60 kDa. As shown in Fig. **2** and Table 2, P450scc protein expression was significantly higher in preeclampsia group than normal pregnancy group (ROD value 6.4940±3.3961 vs. 4.3416±2.8160, P = 0.001). However, when comparing severe preeclampsia group with mild preeclampsia group, there was no significant difference between them (ROD value 6.5712±3.5800 vs. 6.1851±2.6687, P>0.05). The fact that P450scc protein expression in preeclampsia group was significantly higher than normal control indicating that abnormal high expression of CYP11A could contribute to the pathogenesis of preeclampsia.

**Table 2:** p450scc protein expression in normal pregnancy and preeclampsia group relative optical density.

Grouping	The numbers of cases	ROD	P values
Preeclampsia group	59	6.4940±3.3961	0.001
Normal pregnancy group	53	4.3416±2.8160	

t = 3.528; 95% CI (0.9424,3.3623).

### Correlation Analysis of the CYP11A Gene Expression and Clinical Parameters

**Figure 3:**
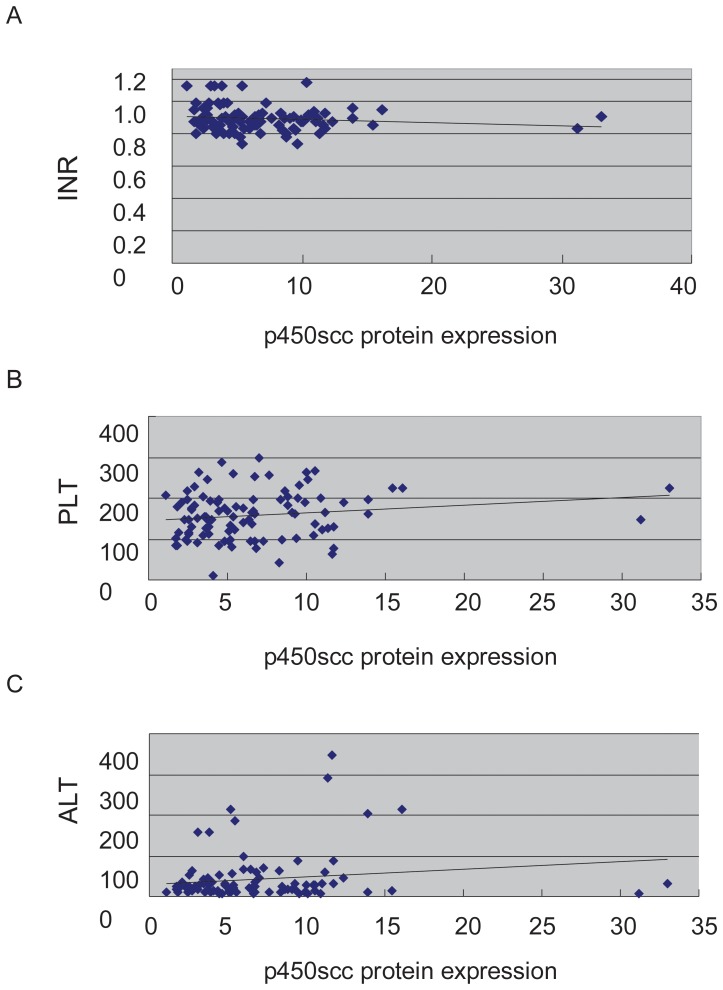
Linear multiple regression result of P450scc protein expression and clinical parameters. (A) CYP11A expression negatively correlated to INR (P = 0.095). (B) CYP11A expression positively correlated to Plt (P = 0.018). (C) CYP11A expression positively correlated to ALT (P = 0.02).

Since CYP11A gene expression and protein levels were significantly upregulated in samples from preeclampsia patients, we then investigated whether clinical parameters of preeclampsia correlated with the altered expression of CYP11A. Univariate analysis was used to determine which parameters were associated with P450scc protein expression (Table 3). All the values with p<0.1 were included in the multiple regression analysis, and the result was shown in table 3.Multiple regression analysis was used to simulate the result and we get the following result: lgP450scc protein = 1.028+0.001ALT+0.001PLT-0.593INR (Fig. 3), indicating that CYP11A gene expression could positively correlated to ALT (Alanine transaminase) level and Plt (platelet level), while negatively correlated to INR (international normalized ratio). The positive correlation indicated that high expression of ALT could be related to dysfunction of liver enzyme, while higher CYP11A gene could be detrimental for the coagulation system.

**Table 3:** p450scc protein expression multiple regression analysis result.

Coefficients^a^						
Model		Unstandardized Coefficients		Standardized Coefficients		
		B	Std. Error	Beta	t	Sig.
1	(Constant)	.693	.035		20.078	.000
	ALT	.001	.000	.211	2.101	.038
2	(Constant)	.497	.090		5.533	.000
	ALT	.001	.000	.257	2.572	.012
	Plt	.001	.000	.235	2.350	.021
3	(Constant)	1.028	.327		3.145	.002
	ALT	.001	.000	.236	2.366	.020
	Plt	.001	.000	.239	2.417	.018
	INR	−.593	.352	−.165	−1.687	.095


a Dependent Variable: lgp450db.

### Overexpression of CYP11A Reduces Cell Viability and Induce Apoptosis in Trophoblast HTR-8/SVneo Cell Lines in Hypoxia Culture Condition

**Figure 4:**
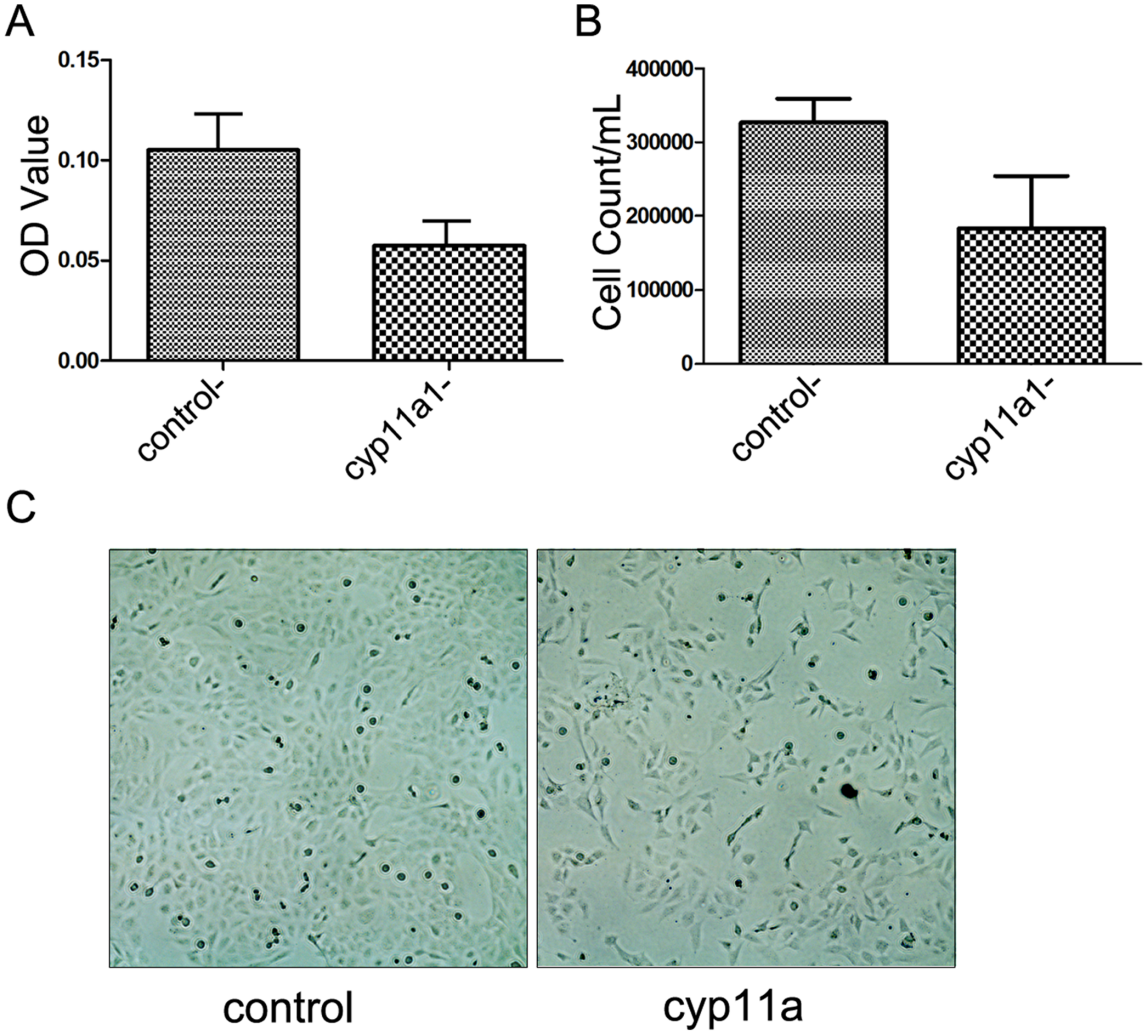
Overexpression of CYP11A reduced HTR-8/SVneo cell viability. (A) Determining the retention of Geimsa stain by cells after 72 h of culture in hypoxia condition. The average optical density (means±SEM) of the Geimsa stain is shown in the bar chart (p<0.05). (B) Cell counts of the CYP11A gene overexpressed and vector control HTR-8/SVneo cell (p<0.05). (C) Representative image of the CYP11A overexpressed (right) and vector control (left) HTR-8/SVneo cells.

**Figure 5:**
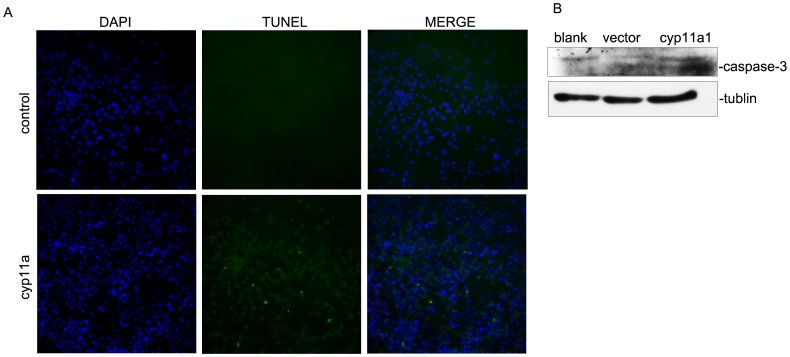
Enhanced apoptosis in CYP11A overexpressed HTR-8/SVneo cells. (A) TUNEL staining shows increased positive signals in CYP11A overexpressed HTR-8/SVneo cells compared to vector control when cultured in hypoxia condition. (B) Western blot result showed increased cleaved caspase-3 expression in CYP11A overexpressed HTR-8/SVneo compared to vector control cells. The image shown is representative result of two independant experiments.

CYP11A has been reported to be involved in regulation of proliferation and apoptosis. We used a trophoblast cell line HTR-8/SVneo and overexpressed CYP11A to determine whether CYP11A affect viability and apoptosis of trophoblast cells. We cultured the cells in hypoxia condition to mimic the hypoxic environment of placentation sites in the uterus during the first 10 weeks of pregnancy. We found that overexpression of CYP11A in HTR-8/SVneo cells significantly reduced cell viability as determined by Giemsa staining (Fig. 4A) and cell counts (Fig. 4B). Furthermore, overexpression of CYP11A resulted in more apoptotic cells when cultured in hypoxia condition as assessed by TUNEL assay, indicating that overexpression of CYP11A indeed could promote apoptosis (Fig. 5A). Caspase-3 is a central executor of mitochondrial dependent apoptosis pathway. To further determine whether the apoptosis promoting effect of CYP11A is dependent on caspase-3 activation, cleaved caspase-3 expression in cultured HTR-8/SV neo cells were evaluated by western blot analysis (Fig. 5B). The results showed that higher level of the 17-kDa cleaved caspase-3 was detected in preeclampsia group as compared to normal pregnancy group (Fig. 5B), suggesting the involvement of caspase-3 activation in the enhanced apoptosis by CYP11A overexpression.

## Discussion

The normal function of the CYP 11A gene is related to cell proliferation, differentiation and apoptosis [22]. By immunohistochemical techniques, we found that the CYP 11A gene is expressed in the cytoplasm of the placental trophoblast cells, consistent with other reports [23]. Western blot showed that the expression of CYP11A was not significantly changed from 1st trimester to full term placenta, suggesting that CYP11 expression in placenta is relatively stable during the pregnancy courses. Since normally only full term placenta is available for most clinical studies, the expression changes of CYP11A from 3rd trimester placenta could reflect expression levels in 1st trimester. This study shows that patients with preeclampsia have significantly increased expression of CYP 11A gene and overexpression of CYP11A in trophoblast cell inhibited trophoblast proliferation and induced apoptosis, thus is possibly involved in the pathological process of preeclampsia. Therefore, for the first time, our result established the link between CYP 11A, a key enzyme in steroid hormone synthesis, and trophoblastic cell behavior, which is integral to the pathogenesis of preeclampsia.

CYP 11A gene may be a candidate gene critical for the development of systemic symptoms of the preeclampsia. In this study, correlation analysis of P450scc expression and the clinical data show that its expression is positively related to ALT, Plt values, while negatively correlated to INR, suggesting that the up-regulated P450scc protein expression may be related to both liver damage and hyper-coagulable state. P450scc is a liver drug metabolizing enzyme involved in human liver exogenous and endogenous compound metabolism. How the increased p450scc protein expressions in patients with preeclampsia lead to liver damage and hypercoagulable state? We speculate that the altered profile of hormone production could explain the links we observed in the study.

How could overproduction of CYP11A lead to abnormal apoptosis? Now it has been known that there are two major pathways in the regulation of apoptosis, and mitochondria play central role in the whole process. Mitochondria have long been recognized as intracellular energy factory; their main role is to provide the necessary energy for a variety of life activities of cells. However, a growing number of studies have found that mitochondria in addition to classic biological effects, also have important role in the regulation of cell survival, in which process mitochondria proteins are critically involved, including changes in the electron transport chain, mitochondrial membrane potential depolarization, oxidation - reduction reactions, etc. CYP 11A encoded protein P450scc is located in the inner mitochondrial membrane and may be one of the key proteins in the regulation of apoptosis in the mitochondria, possibly through activation of caspase-3 pathways. Apoptosis occurs during normal preimplantation embryo development and implantation process, in which trophoblast cells invade the endometrial epithelium and initiate endometrial decidualization and maternal-fetal immune tolerance. Overproduction of CYP11A could promote excessive apoptosis of trophoblast cells, leading to placenta shallow implantation and maternal-fetal immune tolerance imbalance, which contribute to the occurrence of preeclampsia. Whether the observed phenomena that over-expression of CYP 11A gene induced excessive apoptosis of trophoblast cells is related to hormone changes or not is an interesting question warrant further study.

## Supporting Information

Table S1 Clinical characteristics of preeclamptic pregnancies for the analysis of placenta CYP11A expression.
(DOCX)

Table S2 Clinical indicators of preeclamptic pregnancies for the analysis of placenta CYP11A expression
(DOCX)
