# Supplementary material for: Abnormal Apoptosis of Trophoblastic Cells Is Related to the Up-Regulation of CYP11A Gene in Placenta of Preeclampsia Patients
Source: PLoS One. 2013 Mar 29;8(3):e59609. doi: 10.1371/journal.pone.0059609 (PMC3612086; doi:10.1371/journal.pone.0059609)
Supplement: Table S1 — Clinical characteristics of preeclamptic pregnancies for the analysis of placenta CYP11A expression. (DOCX) [file pone.0059609.s001.docx]

**Table S1. Clinical characteristics of preeclamptic pregnancies for the analysis of placenta CYP11A expression**

|  | The number of cases | Age | Gestational age at delivery | Pregnancies | Parity |
| --- | --- | --- | --- | --- | --- |
| **Severe preeclampsia group** | 37 | 31.8±11.4 | 36.7±1.3 | 2.3±0.4 | 1.4±0.5 |
| Mild preeclampsia | 22 | 30.7±7.4 | 38.1±0.3 | 2.3±0.5 | 1.3±0.4 |
| Normal pregnancy group | 53 | 29.8±6.7 | 38.5±0.4 | 2.1±0.7 | 1.2±0.5 |
| P |  | 0.141 0.141 | 0.030 0.030 | 0.712 0.712 | 0.520 |
