# Supplementary material for: Abnormal Apoptosis of Trophoblastic Cells Is Related to the Up-Regulation of CYP11A Gene in Placenta of Preeclampsia Patients
Source: PLoS One. 2013 Mar 29;8(3):e59609. doi: 10.1371/journal.pone.0059609 (PMC3612086; doi:10.1371/journal.pone.0059609)
Supplement: Table S2 — Clinical indicators of preeclamptic pregnancies for the analysis of placenta CYP11A expression (DOCX) [file pone.0059609.s002.docx]

**Table S2. Clinical indicators of preeclamptic pregnancies for the analysis of placenta CYP11A expression**

| Clinical indicators | Severe preeclampsia group | Mild preeclampsia | The normal group | *P* |
| --- | --- | --- | --- | --- |
|  | （37人） (37) | （22人） (22) | （ 53人） (53) |  |
| Incidence of gestational age (weeks) | 31.1±4.5 | 33.0±4.2 | —— - | 0.012 |
| Height (cm) | 158.7±5.6 158.7 ± 5.6 | 158.3 ± 6.1 | 158.1±5.0 | 0.521 |
| Pre-pregnancy weight (kg) | 56.3±7.2 | 55.4±6.6 | 54.7±6.4 | 0.217 |
| Pregnancy weight gain (kg) | 16.1±7.8 | 14.7±6.2 | 12.3 ± 6.2 | 0.000 |
| Birth weight (kg) | 72.8±8.1 | 70.1±5.6 | 67.0±6.0 | 0.000 |
| basic systolic blood pressure | 122.2±8.7 | 109.3±7.9 | 107.1±7.7 | 0.012 |
| Third trimester systolic blood pressure | 155.7±10.6 | 138.1±10.1 | 111.6±6.9 | 0.000 |
| Increased value of Pregnancy systolic blood pressure | 33.5±9.1 | 15.8±4.5 15.8 | 4.5±2.8 | 0.000 |
| The basic of diastolic blood pressure | 87.2±4.8 | 83.2±5.2 | 81.1± 3.7 | 0.679 0.679 |
| Third trimester diastolic blood pressure | 116.9±10.1 | 95.7±7.8 | 82.5±6.1 82.5 ± 6.1 | 0.000 0.000 |
| Pregnancy diastolic blood pressure to increase value | 29.7±7.8 | 12.5±3.4 | 1.4±0.8 1.4 ± 0.8 | 0.000 0.000 |
| HB（g/L） | 109.5±17.9 | 104.4±11.2 | 106.7±12.3 | 0.097 0.097 |
| PLT（×10 ^9^ /L） | 158.6±30.4 | 146.8±29.7 | 159.4±35.8 | 0.296 0.296 |
| PT（s） | 10.2±0.4 | 10.6±0.6 | 10.3±0.5 | 0.675 0.675 |
| APTT（s） | 25.7±2.7 | 24. 9±3.1 | 25.0±2.5 | 0.569 0.569 |
| FIB | 434.1±89.1 | 476.1±90.2 | 467.3±81.2 | 0.102 0.102 |
| ALT（U/L） | 77.0±19.5 | 44.0±15.2 | 22.7±7.8 | 0.000 0.000 |
| AST(U/L) | 83.8±17.9 | 43.7±14.8 | 23.6±7.9 | 0.000 0.000 |
| ALB | 27.1±3.5 | 36.9±2.8 | 39.5±4.1 | 0.001 0.001 |
| UN ( *mmol/L* ) | 5.84 ± 2.18 | 3.01 ± 1.77 | 2.92 ± 0.8 9 | 0.000 0.000 |
| C r ( *μmol/ L* ) | 87.24 ± 21.15 | 41.06 ± 1 0.6 | 44. 1 4 ± 15.0 | 0.000 0.000 |
| Urinary protein excretion *(g/24h)* | 3.45 ± 1.12 | 0.62 ± 0.23 | —— - | 0.000 0.000 |
| Birth weight | 2593.1 ±547.6 | 3020.9 ±342.3 | 3212. ±443.6 | 0.000 0.000 |
